# Brisk walking improves motor function and lower limb muscle strength in Chinese women aged 80 years and older

**DOI:** 10.1038/s41598-024-55925-6

**Published:** 2024-04-04

**Authors:** Yang Wang, Yifan Lu, Zilong Fang, Huiping Yan, Jiahao Li, Zhifan Ye, Yichao Yu, Wei Shan

**Affiliations:** 1 The School of Sports Medicine and Rehabilitation, Beijing Sport University, Beijing, 100084 China; 2 Laboratory of Sports Stress and Adaptation of General Administration of Sport, Beijing Sport University, Beijing, 100084 China; 3 China Institute of Sport and Health Science, Beijing Sport University, Beijing, 100084 China; 4 The School of Sports Coaching, Beijing Sport University, Beijing, 100084 China

**Keywords:** Aging, Metabolomics, Brisk walking, Senior fitness test, Elderly women, Geriatrics, Metabolomics

## Abstract

This study investigates the effects of a 12-week brisk walking exercise regimen on motor function improvements in elderly women. Twenty-six elderly women, aged 84.2 ± 3.2 years, participated in a 12-week brisk walking exercise program. Fitness assessments and blood biomarker analyses (including CHO, HDLC, LDLC, TC) were conducted pre- and post-intervention. Additionally, targeted metabolomics was employed to measure short-chain fatty acids, amino acids, and vitamin metabolites. The intervention led to significant enhancements in participants' flexibility (p < 0.05), lower limb muscle strength (p < 0.01), and cardiorespiratory endurance (p < 0.01), while muscle mass showed no significant changes. Fifteen significant differential metabolites were identified (VIP > 1.0, FC > 1.2 or < 0.8, and p < 0.05), with arginine, ornithine, aspartic acid, glutamine, phenylalanine, tyrosine, and pantothenic acid playing key roles across seven metabolic pathways. A 12-week brisk walking exercise program significantly enhanced flexibility, lower limb muscle strength, and cardiorespiratory endurance among elderly women. These improvements did not extend to muscle mass or upper limb muscle strength. The observed enhancement in exercise capacity may be attributed to improved regulation of neurotransmitters.

## Introduction

Age-related decline in physical function represents a significant health challenge in the elderly population, leading to a diminished capacity for daily living activities and adversely affecting overall quality of life^1^. Mobility impairments often serve as a precursor to functional decline, disability, and frailty, potentially necessitating increased demand for caregiving and medical services^2,3^. Enhancing or preserving physical functionality in elderly individuals is vital not only for maintaining their quality of life and extending healthy lifespans but also for alleviating the strain on healthcare resources. Research has shown that the decline in skeletal muscle mass and function is non-linear, with an accelerated rate of decline occurring around the ages of 75–80^4^. Gender differences are evident, with older women more prone to developing sarcopenia than men^5^. Additionally, older women are at a higher risk of falls and fractures compared to men^6^. In terms of exercise preferences, older adults tend to favor activities that are convenient, low-cost, and can be performed at home^7^. Brisk walking, due to its accessibility, affordability, manageable exercise intensity, and minimal space requirements, is the aerobic exercise most likely to be adopted by older adults^8^. Our previous large-scale questionnaire survey revealed that up to 70% of older adults in China prefer brisk walking as their primary form of daily exercise. Brisk walking involves maintaining a natural gait with the abdomen engaged and the head elevated. The arms swing naturally with elbows slightly bent, and the legs step forward, landing heel-first before rolling onto the forefoot and pushing off. The pace of brisk walking is intermediate between casual walking and race walking.

Recent studies indicate that brisk walking, as a form of exercise, can significantly enhance muscle mass and motor function while concurrently reducing body fat among the elderly. Research conducted by Kubo et al. revealed that 6 months of walking training notably increased muscle thickness and strength in the lower extremities of the elderly^9^. Similarly, Short et al. observed that aerobic exercise boosts muscle protein synthesis across all age groups^10^. Furthermore, Harber et al. identified aerobic exercise training as an effective strategy for augmenting muscle mass and function in older adults^11^. Despite these findings, there has been limited research focused on the very elderly, particularly among Chinese women.

Metabolomics has emerged as a pivotal approach for the systematic analysis of small molecules within biological fluids, cells, and tissues^12^. As metabolites reflect the downstream outcomes of genomic, transcriptomic, and proteomic activities, metabolite-targeted studies are uniquely positioned to elucidate the underlying mechanisms of biological changes. Recent research has highlighted the role of specific amino acids and short-chain fatty acids, metabolites of gut flora, in influencing body composition and exercise capacity. Lustgarten et al.^13^ identified several branched-chain amino acids and novel inflammation-associated tryptophan metabolites as indicators of muscle cross-sectional area or fat-free mass index in functionally limited older adults. Furthermore, Chi-Jen Lo et al.^14^ discovered that the correlation between muscle mass and metabolites in older adults is gender-specific, with muscle mass in older women being associated with glutamate levels. Beyond amino acids, short-chain fatty acids produced in the gut exhibit various positive effects on energy metabolism, including direct impacts on peripheral tissues, enhancement of lipid metabolism^15^, and reduction of cardiovascular risk^16^. Animal studies have demonstrated that butyrate, a short-chain fatty acid, positively influences muscle mass in aging^17^. Nonetheless, the majority of these findings stem from cross-sectional studies, and there is a notable scarcity of longitudinal intervention studies involving older adults.

The aim of this study was to explore the association between alterations in body composition and motor function and changes in metabolites and metabolic pathways in elderly women following a 12-week walking exercise intervention. This investigation employed targeted metabolomics to furnish a theoretical foundation for formulating precise exercise prescriptions for elderly women.

## Materials and methods

### Research design and subjects

This study employs a quasi-experimental design to examine the impact of a 12-week program of moderate-intensity brisk walking, supervised by researchers, on motor function and metabolites including short-chain fatty acids, amino acids, and vitamins in women aged over 80 years. Conducted in Langfang, Hebei Province, the research recruited a cohort of 26 oldest-old female adults from a nursing home, with an average age of 84.2 ± 3.2 years. Inclusion criteria were: (1) females over 80 years of age capable of walking independently; (2) classified as low-to-moderate cardiovascular risk according to ACSM guidelines^18^; (3) consented to participate in the 12-week brisk walking program and provided signed informed consent. Exclusion criteria included: (1) individuals with coronary stents, pacemakers, joint replacements, or fractures within the last 3 months; (2) those suffering from diabetes, arthritis, or any condition inhibiting normal exercise; (3) those diagnosed with asthma, malignancy, chronic heart failure, cancer, severe depression, or other psychiatric disorders; (4) those with a participation rate in the exercise program below 75%. Data were collected through questionnaires, which were administered and scored by trained investigators.

All participants were fully briefed on the procedures, aims, and potential risks associated with the study, providing written informed consent prior to participation. The study received approval from the Ethics Committee of Beijing Sport University and adhered to the ethical standards laid out in the Declaration of Helsinki. A general questionnaire gathered information on age, gender, and medical history.

### Collection of test parameters

#### Body composition indicators

Height was assessed using a standardized height meter. Participants' weight and body composition were determined using INBODY230. Participants were instructed to refrain from vigorous exercise for 48 h, and from consuming coffee or alcohol for 24 h prior to the body composition assessment. They were also advised to urinate 30 min before the test and to remove any excess clothing during the measurement. The appendicular skeletal muscle mass index (ASMI) and body mass index (BMI) were calculated based on these measurements.

$$\begin{aligned} & {\text{ASMI }} = {\text{ Appendicular Skeletal Muscle Mass}}\left( {{\text{kg}}} \right)/{\text{Height}}^{{2}} \left( {{\text{m}}^{{2}} } \right) \\ & {\text{BMI}} = {\text{ Weight }}\left( {{\text{kg}}} \right)/{\text{ Height}}^{{2}} \left( {{\text{m}}^{{2}} } \right) \\ \end{aligned}$$

### Index of motor function

Participants conducted the designated tests without reliance on assistive devices, under the supervision of at least one researcher positioned adjacent to them to prevent falls. Table 1 outlines the procedures for all motor function assessments, along with their specific objectives.

**Table 1:** The procedure for all motor function tests and the purposes of the tests.

Test	Procedure	Purpose of the test
Handgrip strength	Measured using an electronic dynamometer (Jamar) to measure the grip strength of the subject's dominant hand, with the maximum value taken twice	Assessing upper extremity muscle strength. The recommended assessment of muscle strength in the EWGSOP2
Chair sit and reach test	The height of the chair was 43 cm, the subject was seated at the front end of the chair with the dominant leg straight in front, arms folded, leaning over to reach or exceed the toes as far as possible with the hands, and the distance from the fingertips to the toes was measured, and the maximum value was taken on two occasions	Assessing hamstring flexibility
4-m gait speed	The subject walked 4 m at their usual walking pace, calculate the walking distance per second, test twice and recorded the fastest performance	Assessing walking stability
2-min step test	Participants were instructed to lift their legs alternately to a specified height as much as possible within 2 min, with each knee reaching the marked height counted as one repetition	Assessing aerobic endurance function
Timed Up and Go test	The subject sits in a reclining chair with armrests and leans on the back of the chair and tests the time it takes to stand up from a seated position, walk forward 3 m, turn around and walk back to the front of the chair, turn around again and sit down and lean on the back of the chair, and averages the two times the test is performed	Assessing balance and coordination skills besides walking stability
30-s sit-to-stand test	Requires the subject to sit in a 45-cm-high, straight-backed chair, and to complete as many of the get-up-sit-down movements as possible in 30 s	Assessing lower extremity muscle strength

### The exercise intervention program

All participants adhered to the exercise intervention protocol outlined in the ACSM's Guidelines for Exercise Testing and Prescription, Tenth Edition^18^. The regimen included brisk walking at moderate intensity, targeting a heart rate reserve of 40–60%, conducted three times a week (on alternate days) for 45 min per session. Each session was preceded and followed by 5 min of warm-up and cool-down activities, respectively, which were not counted towards the 45-min exercise duration. The intervention was overseen by trained researchers, with participants' heart rates during exercise monitored via sports watches. These watches were programmed to emit an alarm to notify the supervisor if a participant's heart rate deviated from the predefined zone, enabling timely adjustments (either to decelerate or accelerate). Participants with an absenteeism rate exceeding 25% were considered to have withdrawn from the study.

### Biological sample collection and testing

Fasting venous blood samples were drawn from the brachial vein between 6:00 and 7:00 a.m. Upon collection, whole blood was centrifuged at 1000 rpm for 10 min at room temperature to isolate the serum. The serum samples were then promptly frozen at − 80 °C pending further analysis for blood testing or metabolomics studies. Participants collected fecal samples during their first bowel movement of the day. These samples were preserved with a kit containing a stabilizer effective at room temperature, allowing for storage at room temperature for up to two weeks for fecal metagenomic analysis.

### Biomarker testing

Biomarker assays were conducted utilizing a Beckman Coulter DxC800 automated biochemistry analyzer and Leadman's reagent kit. On the testing day, samples were retrieved from − 80 °C storage and gradually thawed until they reached room temperature. Following calibration of the instrument with lyophilized standards, the biomarker assays were executed in accordance with established quality control protocols.

### Metabolomics testing and analysis

To mitigate errors across batches, samples collected before and after the intervention were analyzed concurrently. Metabolomic analyses were conducted by BGI Genomics in Shenzhen. The LC–MS/MS analyses utilized the TranscendII-Sciex5500 (Waters, UK) and Waters Iclass-AB Sciex 6500 (Waters, UK) liquid chromatography-mass spectrometry systems to assess short-chain fatty acids, amino acids, and vitamin. Data analysis was carried out using MetaboAnalyst (https://genap.metaboanalyst.ca/MetaboAnalyst/). Pareto scaling and sample median normalization techniques standardized the data. The analysis employed principal component analysis (PCA) and orthogonal partial least squares discriminant analysis (OPLS-DA) for model construction, with permutation tests to evaluate overfitting. Differential metabolites identified pre- and post-exercise intervention were determined through two-tailed paired T-tests, applying false discovery rate (FDR)-adjusted p-values, fold change (FC) values, and variable importance in projection (VIP) values from the OPLS-DA model on normalized raw data. Metabolites with VIP > 1.0, FC > 1.2 or < 0.8, and P < 0.05 were deemed statistically significant. These metabolites underwent pathway enrichment and topology analysis using the KEGG database through the MetaboAnalyst platform to elucidate pathways influenced by the brisk walking intervention in elderly women. Topological analysis of differential metabolites utilized relative-betweenness centrality.

### Statistical analysis

Statistical analysis was conducted using Python version 3.9. The Shapiro–Wilk test assessed the normality of data distributions. For continuous variables adhering to a normal distribution, outcomes were expressed as "mean ± standard deviation," with paired sample t-tests (α < 0.05) employed to evaluate differences pre- and post-intervention. Non-normally distributed data or count variables were reported as median (interquartile range), and the Wilcoxon rank-sum test (α < 0.05) was utilized for comparison between groups. A p-value of less than 0.05 was deemed indicative of statistical significance.

### Institutional Review Board Statement

The study was approved by the Ethics Committee of Beijing Sport University, approval No. 2020082H.

## Results

### Body composition and exercise performance before and after the intervention

Table 2 presents the outcomes of various metrics evaluated before and after the brisk walking intervention among participants. Following the 12-week program, there was a notable increase in body weight, body fat percentage, and Body Mass Index (BMI). Conversely, the Appendicular Skeletal Muscle Mass Index (ASMI) did not exhibit significant alterations. In terms of motor function assessments, enhancements were observed in the chair sit and reach distance (p < 0.05), 4-m gait speed (p < 0.01), and the number of repetitions in the 2-min step test (p < 0.01), all showing significant improvements. The time required for the 30-s sit-to-stand test decreased substantially (p < 0.05). Although grip strength demonstrated some improvement, the variation was not statistically significant. Similarly, improvements were noted in the Timed Up and Go Test (TUGT) and the Short Physical Performance Battery (SPPB) scores, yet these did not achieve statistical significance. Post-intervention, Low-Density Lipoprotein Cholesterol (LDL-C) levels significantly diminished, the ratio of High-Density Lipoprotein Cholesterol (HDL-C) to LDL-C significantly escalated, and levels of total cholesterol, HDL, and triglycerides experienced no significant changes.

**Table 2:** Subjects' body composition, exercise test results and blood biomarkers before and after the intervention.

Parameters	Pre	Post	p value
Weight (kg)	53.74 ± 8.53	54.52 ± 8.19	0.02
Body fat (%)	31.50 ± 6.52	33.06 ± 6.29	< 0.01
BMI (kg/m^2^)	22.10 ± 3.05	22.47 ± 2.92	< 0.01
ASMI (kg/m^2^)	5.51 ± 0.68	5.50 ± 0.68	0.86
HGS (kg)	18.36 ± 3.45	19.13 ± 2.98	0.06
CSRT (cm)	2.72 ± 6.85	6.17 ± 5.67	0.03
4MGS (m/s)	1.10 ± 0.24	1.26 ± 0.21	< 0.01
TUGT (s)	11.29 ± 1.63	10.77 ± 1.43	0.14
30SST (reps)	12.27 ± 3.63	14.00 ± 3.59	< 0.01
2MST (reps)	70.08 ± 22.22	80.46 ± 21.84	< 0.01
CHO (mmol/L)	4.61 ± 1.06	4.22 ± 0.89	0.19
HDL-C (mmol/L)	1.47 ± 0.24	1.61 ± 0.39	0.16
LDL-C (mmol/L)	2.77 ± 0.93	2.27 ± 0.74	0.04
HDL-C/LDL-C	0.33 ± 0.07	0.39 ± 0.09	0.03
TG (mmol/L)	1.31 ± 0.47	1.29 ± 0.81	0.95

Data are presented as “mean ± SD” or median (upper quartile, lower quartile).

*BMI* Body Mass Index, *ASMI* Appendicular Skeletal Muscle Mass Index, *HGS* handgrip strength, *CSRT* chair sit and reach test, *TUGT* Timed Up and Go Test, *30SST* 30-s sit-to-stand test, *4MGS* 4-m gait speed, *2MST* 2-min step test, *CHO* total cholesterol, *HDL-C* high-density lipoprotein, *LDL-C* low-density lipoprotein, *TG* triglycerides.

### Targeted metabolomics analysis before and after intervention

**Figure 1:**
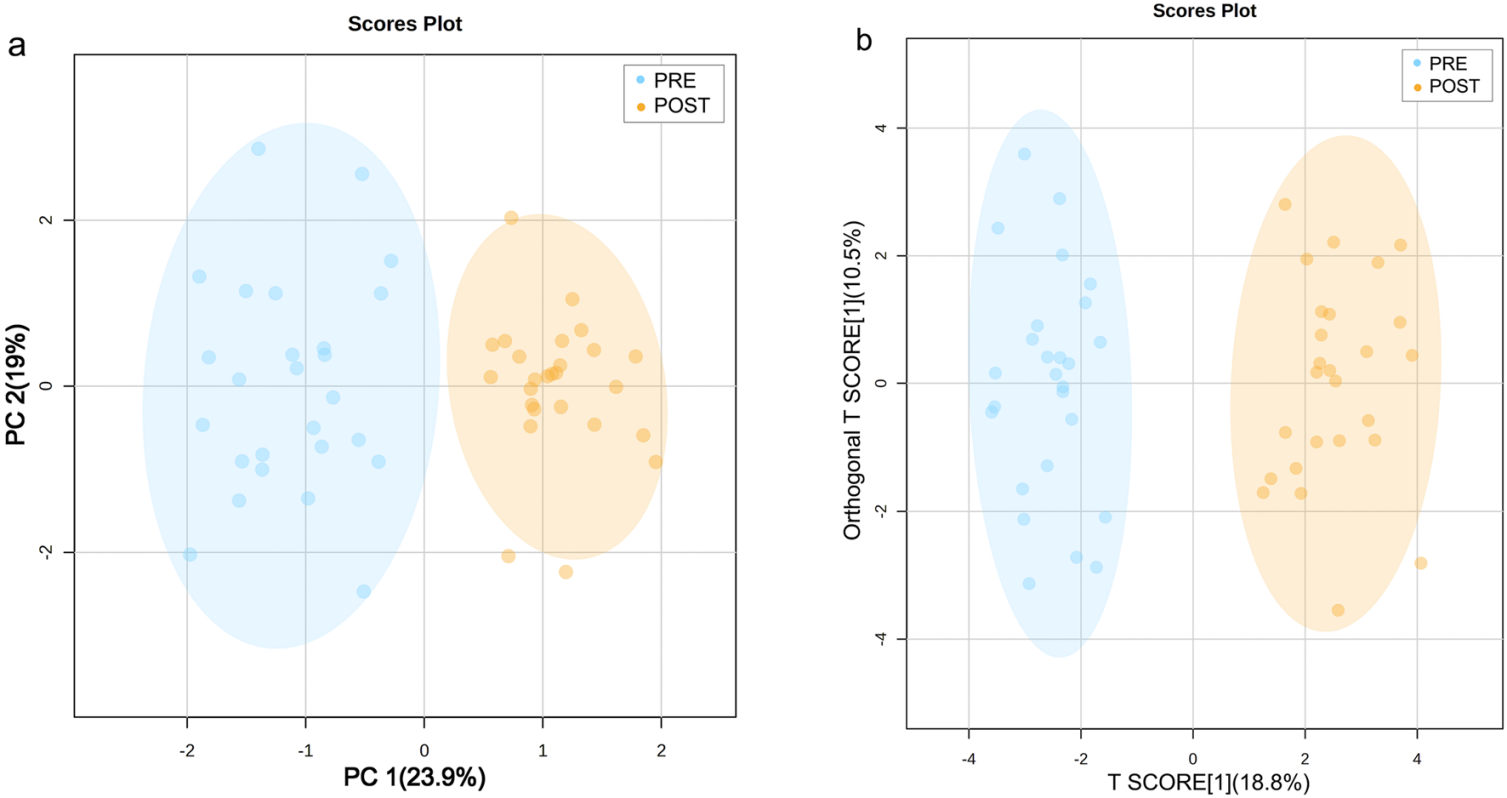
(**a**) Principal component analysis (PCA) plot of plasma metabolites in subjects; (**b**) orthogonal Partial Least Squares Discriminant Analysis (OPLS-DA) plot of plasma metabolites in subjects.

The PCA of plasma metabolites before and after the intervention is depicted in Fig. 1a. There is a distinct separation between baseline and post-intervention samples in the principal component, signifying metabolic alterations in participants due to the intervention. The PCA accounted for approximately 23.9% of the variance with the first eigenvalue and about 19% with the second eigenvalue.

**Figure 2:**
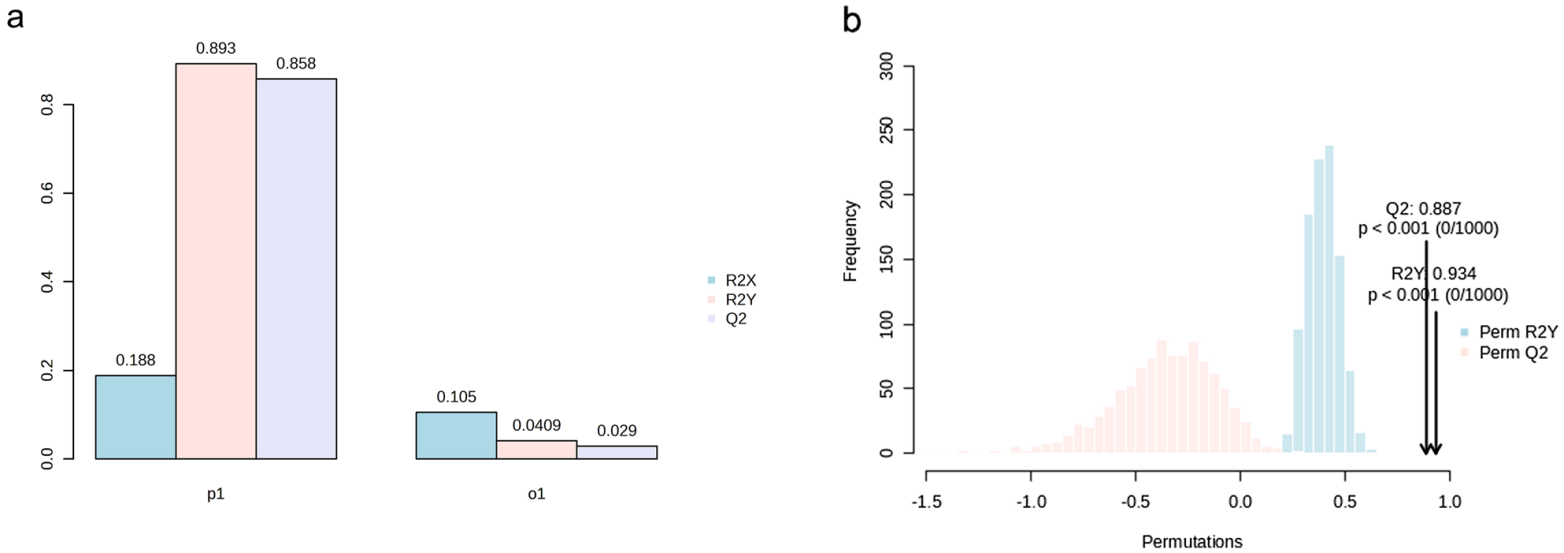
(**a**) Parameters of the OPLS-DA prediction model. (**b**) Permutation test results of the OPLS-DA model.

A supervised OPLS-DA demonstrated a clear demarcation trend between baseline and post-intervention samples, with no overlap observed between groups (Fig. 1b). The model's parameters, R2Y, was 0.893, and Q2 was 0.858 (Fig. 2a). The permutation test results (Fig. 2b) confirmed the absence of overfitting, establishing the model's stability, reliability, and robust predictive capability.

**Figure 3:**
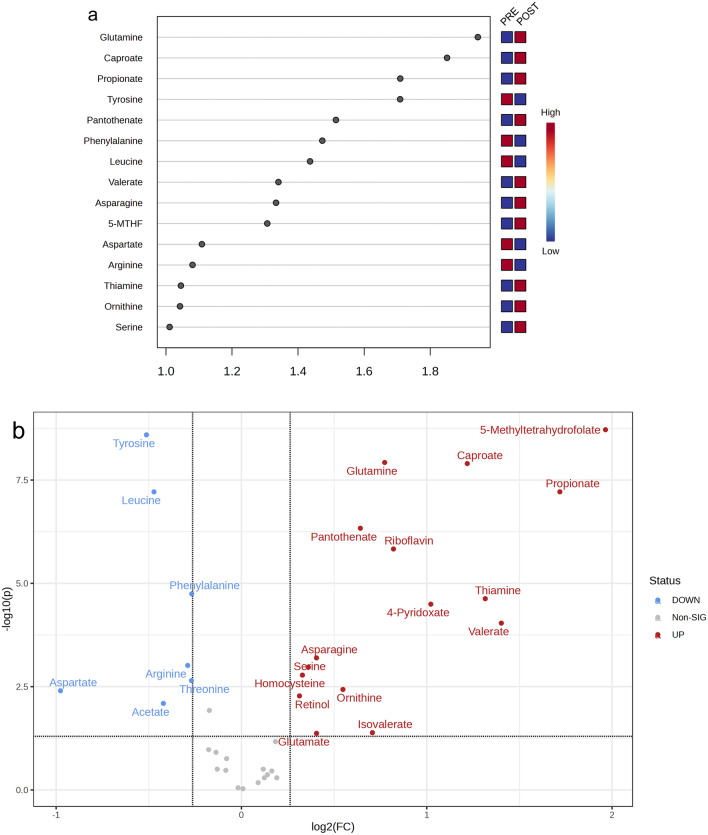
(**a**) Variable importance in projection (VIP) plot of the OPLS-DA Model. 5-MTHF: 5-Methyltetrahydrofolate; (**b**) Volcano plot of metabolites significantly altered after the intervention. Fold change (FC) values of 1.2 or 0.8 and significance level of p < 0.05, with the X-axis and Y-axis representing log-transformed FC values and p-values. The results have been corrected for False Discovery Rate (FDR).

Both PCA and OPLS-DA plots underscored that the exercise intervention prompted metabolic changes in the subjects. Figure 3a,b, and Table 3 reveal that targeted metabolomics identified a total of 15 differential metabolites (VIP > 1, FC > 1.2 or < 0.8, p < 0.05), indicating significant metabolic shifts as a result of the intervention.

**Table 3:** Significantly differential metabolite.

Nos.	Metabolite	Class	FC	Change	p adjusted
1	Tyrosine	Amino acid	0.7	Downregulated	4.57 × 10^9^
2	Propionate	Short chain fatty acid	3.3	Upregulated	1.13 × 10^8^
3	Caproate	Short chain fatty acid	2.3	Upregulated	1.13 × 10^8^
4	Glutamine	Amino acid	1.7	Upregulated	1.13 × 10^8^
5	5-MTHF	Water-soluble vitamins	3.9	Upregulated	1.45 × 10^8^
6	Leucine	Amino acid	0.7	Downregulated	7.70 × 10^8^
7	Pantothenate	Water-soluble vitamins	1.6	Upregulated	4.68 × 10^7^
8	Phenylalanine	Amino acid	0.8	Downregulated	1.76 × 10^5^
9	Thiamine	Water-soluble vitamins	2.5	Upregulated	6.18 × 10^5^
10	Valerate	Short chain fatty acid	2.6	Upregulated	6.89 × 10^5^
11	Asparagine	Amino acid	1.3	Upregulated	6.19 × 10^4^
12	Arginine	Amino acid	0.8	Downregulated	9.32 × 10^4^
13	Serine	Amino acid	1.3	Upregulated	1.31 × 10^3^
14	Aspartate	Amino acid	0.5	Downregulated	3.33 × 10^3^
15	Ornithine	Amino acid	1.5	Upregulated	5.51 × 10^3^

*5-MTHF* 5-Methyltetrahydrofolate, *FC* fold change, *p adjusted* the p value has been corrected for False Discovery Rate (FDR).

### Quantitative enrichment analysis of differential metabolites and pathway topology analysis

**Figure 4:**
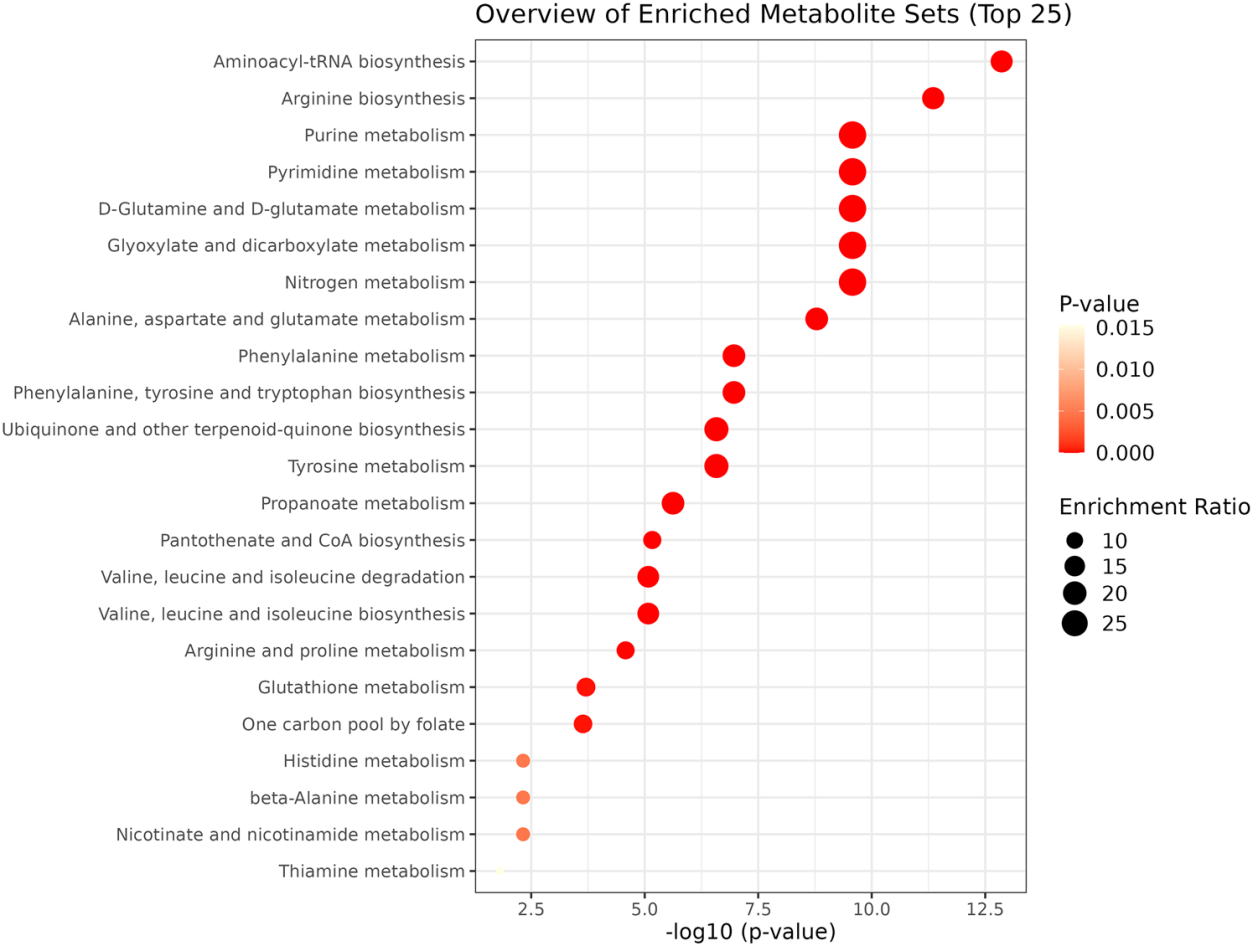
Results of enrichment analysis. Enrichment ratio is computed by hits/expected, where hits = observed hits; expected = expected hits.

Quantitative enrichment analysis, as depicted in Fig. 4, revealed that the metabolic profile differences between subjects before and after the intervention predominantly enriched metabolite groups in pathways such as aminoacyl-tRNA biosynthesis, arginine biosynthesis, purine metabolism, pyrimidine metabolism, D-glutamine and D-glutamate metabolism, glyoxylate and dicarboxylate metabolism, nitrogen metabolism, alanine, aspartate and glutamate metabolism, phenylalanine metabolism, aromatic amino acid biosynthesis, ubiquinone and other terpenoid-quinone biosynthesis, and tyrosine metabolism. These pathways are principally engaged in nitrogen metabolism and the synthesis and metabolism of neurotransmitters.

The intervention's impact, as influenced by the 15 differential metabolites, was evident across 23 pathways (Table 4). Notably, seven pathways—arginine biosynthesis; alanine, aspartate, and glutamate metabolism; aromatic amino acid synthesis; phenylalanine metabolism; tyrosine metabolism; pantothenate and coenzyme A biosynthesis; and arginine and proline metabolism—were significantly affected by the intervention (impact value > 0), indicating a substantial influence on metabolic changes post-intervention.

**Table 4:** Pathway topology analysis results.

Pathway	Matched metabolites	p value^a^	Impact^b^
Phenylalanine, tyrosine and tryptophan biosynthesis	Phenylalanine; tyrosine	2.49 × 10^7^	1
Phenylalanine metabolism	Phenylalanine; tyrosine	2.49 × 10^7^	0.35714
Alanine, aspartate and glutamate metabolism	Asparagine; aspartate; glutamine	4.68 × 10^9^	0.33734
Arginine and proline metabolism	Arginine; ornithine	3.56 × 10^5^	0.16849
Tyrosine metabolism	Tyrosine	5.05 × 10^7^	0.13972
Arginine biosynthesis	Arginine; aspartate; ornithine; glutamine	5.08 × 10^11^	0.13705
Pantothenate and CoA biosynthesis	Pantothenate; aspartate	1.12 × 10^5^	0.00714
Aminoacyl-tRNA biosynthesis	Asparagine; phenylalanine; arginine; glutamine; aspartate; leucine; tyrosine	3.17 × 10^12^	0
Purine metabolism	Glutamine	8.69 × 10^10^	0
Pyrimidine metabolism	Glutamine	8.69 × 10^10^	0
D-Glutamine and D-glutamate metabolism	Glutamine	8.69 × 10^10^	0
Glyoxylate and dicarboxylate metabolism	Glutamine	8.69 × 10^10^	0
Nitrogen metabolism	Glutamine	8.69 × 10^10^	0
Ubiquinone and other terpenoid-quinone biosynthesis	Tyrosine	5.05 × 10^7^	0
Propanoate metabolism	Propanoate	4.21 × 10^6^	0
Valine, leucine and isoleucine degradation	Leucine	1.20 × 10^5^	0
Valine, leucine and isoleucine biosynthesis	Leucine	1.20 × 10^5^	0
Glutathione metabolism	Ornithine	2.51 × 10^4^	0
One carbon pool by folate	5-Methyltetrahydrofolate	2.76 × 10^4^	0
Histidine metabolism	Aspartate	4.98 × 10^3^	0
beta-Alanine metabolism	Aspartate	4.98 × 10^3^	0
Nicotinate and nicotinamide metabolism	Aspartate	4.98 × 10^3^	0
Thiamine metabolism	Thiamine	1.53 × 10^2^	0

^a^p-values from pathway enrichment analysis.

^b^Impact values from pathway topology analysis.

## Discussion

This research, utilizing LC–MS-targeted metabolomics, entailed a 12-week brisk walking exercise regimen for 26 women aged over 80 years. Evaluations were conducted both before and after the intervention, examining motor function, blood biochemical markers, and plasma metabolites. Subsequent to the 12-week exercise program, significant alterations were noted in the participants' body composition, motor function, and plasma metabolite profiles.

### Changes of motor function after brisk walking intervention

The chair-sit-and-reach test evaluates lower limb flexibility, underscoring the efficacy of pre-exercise warm-ups and post-exercise cool-downs in enhancing flexibility among older adults. This observation aligns with findings from Parkatti et al.^19^ and Kortas et al.^20^. Post-intervention assessments of the 30-s sit-to-stand, 4-m gait speed, and 2-min step test revealed significant enhancements, corroborating with prior research^9,11,19,21,22^. These improvements suggest enhancements in lower limb muscle strength and mobility, as well as in muscular endurance and cardiorespiratory fitness. The observed benefits likely stem from the walking exercise's demand for integrated hip-leg force generation, which bolsters lower limb strength. Concurrently, walking, as an aerobic activity, effectively fosters mitochondrial function enhancement^23^ and boosts aerobic endurance and muscular function^11^.

The Timed Up and Go Test (TUGT) exhibited some improvement post-intervention, although the effect did not reach statistical significance. This outcome may be attributed to the complexity of the functional indices measured by the TUGT, which, besides muscle strength, also depend on balance and coordination abilities^24,25^. These abilities are influenced by various factors including vestibular function, proprioception, vision, and neuromuscular regulatory sensitivity. The walking exercise regimen and environment provided limited stimulation to lower limb proprioceptors and vestibular function, thereby constraining enhancements in balance and coordination. To augment improvements in these areas, it is suggested that the walking environment be modified to increase stimulation of vestibular and proprioceptive systems, such as by walking on uneven surfaces or using different road materials, while ensuring safety precautions are adhered to.

Contrary to the findings of several studies^26–28^, participants in our study exhibited significant increases in body weight, body fat percentage, and BMI post-intervention, which could be linked to enhanced nutritional status. Cheng Q et al.^29^ posited that in older adults, a higher body fat percentage suggests greater protein intake. A cross-sectional analysis of Chinese older adults revealed a significant, positive correlation between BMI and health utility scores as well as activity levels, indicating that a higher BMI might serve as a protective health factor in this demographic^30^. Moreover, the reduction in Low-Density Lipoprotein Cholesterol (LDL-C) levels and the elevation in the HDL-C to CHO ratio post-intervention underscore the positive impact of walking exercise on cardiovascular health in older women^31^, even amidst an increase in body fat percentage.

### Analysis of plasma metabolites after brisk walking intervention

Following the 12-week walking intervention, analysis of the plasma metabolites identified 15 differential metabolites. Subsequent topological analysis highlighted seven pathways with significant post-intervention impact: arginine biosynthesis; alanine, aspartate, and glutamate metabolism; aromatic amino acid synthesis; phenylalanine metabolism; tyrosine metabolism; pantothenate and CoA biosynthesis; and arginine and proline metabolism.

Post-intervention, levels of phenylalanine and tyrosine in participants were significantly reduced. Both phenylalanine and tyrosine are aromatic amino acids, with phenylalanine serving as a precursor to tyrosine. Tyrosine is synthesized from phenylalanine through the action of tyrosine hydroxylase. Further, tyrosine is converted into 3,4-dihydroxyphenylalanine by tyrosine hydroxylase and subsequently transformed into vital neurotransmitters and hormones such as dopamine, norepinephrine, and epinephrine. These compounds play crucial roles in regulating functions of the central nervous system and autonomic nervous system. Research indicates plasma phenylalanine levels increase with age^32^ and exhibit a negative correlation with leukocyte telomere length^33^, a biomarker of aging^34^. Moreover, phenylalanine and tyrosine concentrations have been positively associated with the risk^35^ and severity^36^ of heart failure, and inversely related to coenzyme Q10 levels^37^, which is essential for maintaining mitochondrial function and promoting cardiovascular health. The notable increase in 2-min step test counts among participants post-intervention suggests an amalgamated effect of enhanced mitochondrial function, cardiovascular health, and reduced catecholamine concentrations.

Glutamine, the most abundant amino acid in the human body, serves as the principal transporter of NH4^+^ in the bloodstream. It directly supplies nutrients for nucleotide biosynthesis and indirectly contributes carbon and nitrogen for pyrimidine biosynthesis. As a crucial intermediary in the TCA cycle, glutamine plays a pivotal role in energy metabolism. It is posited that glutamine metabolism is a downstream process of the activation of the mTOR pathway^38^, with mTOR partially enhancing energy production by activating glutamate dehydrogenase, facilitating the conversion of glutamate to glutamine^39^. The notable post-intervention rise in blood glutamine concentration among participants might signify an improved nutritional status and activation of the mTOR pathway. Contrary to the findings of several studies^26–28^, this investigation observed significant post-intervention increases in body weight, body fat percentage, and HDL-C/LDL-C ratio. A higher body fat percentage in older adults is associated with increased protein intake^29^. The absence of significant changes in skeletal muscle mass among the subjects could be attributed to their advanced age, elevated catabolic rate, and the insufficiency of the anabolic rate increase to effect a rise in skeletal muscle mass.

A cross-sectional analysis of Taiwanese older adults revealed that the ornithine/arginine ratio was elevated in individuals with high skeletal muscle mass compared to those with lower skeletal muscle mass^14^. Arginine, a crucial intermediary in the urea cycle, displayed a significant positive correlation with age. In our study, there was a notable increase in ornithine levels, whereas arginine levels were significantly decreased post-intervention, leading to an increased ornithine/arginine ratio. These findings suggest that brisk walking exercise effectively mitigates muscle loss in the elderly, potentially exerting an anti-aging effect concurrently.

The association between aspartate levels and aging continues to be debated. Certain studies indicate a positive correlation between blood aspartate concentrations and aging^32,40^. Conversely, other research suggests that with advancing age, catabolic processes intensify, leading to elevated blood urea levels, which in turn, due to increased urinary excretion, should result in reduced aspartate concentrations in the body^41^. In summary, the relationship between aspartate and aging, including the underlying mechanisms, requires further investigation.

Pantothenate primarily functions to synthesize coenzyme A and acyl carrier proteins, facilitating various metabolic processes. Coenzyme A is transformed into acetyl-coenzyme A by pyruvate dehydrogenase^42^, a critical substrate for the citric acid cycle. Furthermore, pantothenate contributes to the biosynthesis of steroid hormones and acetylcholine in the brain through acetyl-CoA. A deficiency in pantothenate, a vital precursor for acetylcholine production, may result in reduced acetylcholine levels, leading to cognitive and motor impairments. Lower levels of pantothenate have been noted in individuals diagnosed with Alzheimer's, Parkinson's, and Huntington's diseases in comparison to healthy subjects^43–45^. Post-intervention, participants in this study likely experienced enhanced nutritional status, manifesting in a significant rise in plasma pantothenic acid levels, increased brain acetyl-CoA and acetylcholine concentrations, and improved motor and cognitive functions.

Our study presents several limitations: Firstly, the employed targeted metabolomics approach might not capture all potential differential metabolites. Secondly, the absence of strict nutrition and medication control during the intervention could influence the outcomes. Thirdly, the lack of a control group in the experiment may impact the interpretation of the intervention's effectiveness. Lastly, given this research is focused on exercise intervention, individuals unable to participate in such activities were not included at the recruitment phase, suggesting that the applicability of our findings to broader elderly populations should be approached with caution. Future studies should encompass diverse health conditions. Despite these limitations, this research stands as the inaugural metabolomics-based examination into the mechanisms through which brisk walking enhances exercise functionality, delving into the metabolic and pathway alterations contributing to exercise function improvement in Chinese elderly women. This provides a foundational theoretical basis for the refined development of exercise prescriptions tailored for elderly women.

## Conclusion

In conclusion, a 12-week regimen of brisk walking significantly enhances lower limb flexibility, muscle strength, muscle endurance, cardiopulmonary function, and lipid metabolism in the oldest-old women. The metabolic pathways of aromatic amino acids, aspartate and glutamate, arginine and purine, along with pantothenic acid and coenzyme A biosynthesis, are linked to improvements in muscle strength and motor function. The observed enhancements in muscle strength and motor function among the oldest-old women are attributed primarily to changes in neurotransmitters within the body, rather than an increase in muscle mass.

## Data Availability

The data of the current study are available from the corresponding author upon reasonable request.
